# Nonpharmacological Treatment for Supporting Social Participation of Adults with Depression

**DOI:** 10.1155/2021/8850364

**Published:** 2021-04-29

**Authors:** Supaluck Phadsri, Rieko Shioji, Atsuko Tanimura, Jeerawit Jaknissai, Sopida Apichai, Tippawan Sookruay

**Affiliations:** ^1^Department of Occupational Therapy, Tokyo Metropolitan University, Tokyo 116-8551, Japan; ^2^Department of Occupational Therapy, Chiang Mai University, Chiang Mai 50200, Thailand; ^3^Occupational Therapy Unit, Thanyarak Khon Kaen Hospital, Khon Kaen 40000, Thailand; ^4^Chiang Mai University Library, Chiang Mai University, Chiang Mai 50200, Thailand

## Abstract

**Background:**

Social withdrawal is predominantly seen among adults with depression. However, a dearth of reviews exists that explore nonpharmacological treatments, especially occupational therapy (OT) interventions and their effect in promoting social participation. The aim of this research was to review what intervention programs are conducted to support the social participation of adults with depression and their effectiveness.

**Method:**

A systematic review was performed wherein relevant articles were searched in PubMed, CINAHL, Wiley Online Library, PsycINFO, and OTseeker databases and AJOT, BJOT, SJOT, and OTMH journals. Only English articles published from January 2010 to December 2018, which tackled intervention for adults aged 20–60 years with depression, were considered. Ten out of 918 studies met the screening criteria.

**Result:**

Among the ten studies, the effective intervention programs were categorized as either occupation-based intervention (OBI) or cognitive behavioral therapy-based intervention (CBT-BI). These programs sought the following outcomes: behavioral change in social participation (*n* = 4), reduction of depression or depressive symptoms (*n* = 13), life satisfaction (*n* = 4), and quality of life (QoL) (*n* = 1). Studies showed moderate (*n* = 3) to strong (*n* = 7) level of certainty, whereas they also revealed high to unclear (*n* = 3) and low (*n* = 7) risk of bias.

**Conclusion:**

Both OBI such as animal-assisted therapy and CBT-BI such as behavioral change program and health education have a strong level of certainty and low risk of bias in promoting social participation by supporting positive behavioral change and reducing depressive symptoms. Furthermore, the sport and exercise program of OBI was popular in encouraging participation and engagement with other people. Other programs were suggested for combined interventions to support social participation, life satisfaction, and QoL.

## 1. Introduction

Depression is a global concern and is predicted to become the leading, serious, and chronic noncommunicative disease by 2030 [1]. Depression is frequently revealed by an individual's poor social experience and impaired social functioning [2]. Furthermore, depression may produce long-term behavioral change by increasing social avoidance [3, 4], which has an impact on one's occupation, especially social participation [5]. As people with depression are overwhelmed with negative thoughts and have a lesser drive to participate in social activities, they are at a high risk of experiencing a relapse of symptoms, resulting in diminished self-love, life satisfaction, and quality of life (QoL) [6]. The importance of occupational performance and social participation is supported and covered by activity and participation in the World Health Organization's International Classification of Functioning, Disability, and Health (ICF) [7].

Social participation is defined in the occupational therapy profession as “the interweaving of occupations to support desired engagement in community and family activities as well as those involving peers and friends,… and that support social interdependence” [5]. As social participation plays an essential role in recovering from depression, occupational therapy practitioners should deliver occupation-focused interventions that “support the development of relationship and companionship with peers, friends, partners, and pets, which are very important in social participation” [8].

The literature review revealed that nonpharmacological treatment is the preferred treatment option by the majority of people with depression [4, 9]. The negative perception of pharmacological treatment [4] among this group is mainly due to the unpleasant side effects they experienced, such as drowsiness, weight gain, fatigue, constipation, and sexual dysfunction [10, 11], which often led to inadequate work capacity and loss of social functioning [6]. Furthermore, receiving pharmacological treatment may demand special supervision from other people, which could be a burden for the family and community [12]. Thus, even if they agreed to use antidepressants for retaining functional ability [11], the promotion of social participation with nonpharmacological treatment is still required in order to assist in reducing depressive symptoms [13, 14] and enrich social interaction, social identity, participation, and sense of belonging [15]. These benefits, in turn, provide opportunities to improve life satisfaction and QoL [6, 8].

To manage depression and improve mental and social health, individuals with depression need to work themselves and cooperate with other people in the community and society. Therefore, supportive social participation and rehabilitation processes of adults with depression are typically motivated by the recovery and active management of their illness in terms of personal and social well-being [8]. While several reviews about social participation have been published, these reviews looked into older adults and focused on social participation as a subconstruct of leisure [16, 17] or considered insufficient studies with a low level of evidence [18]. In realizing the importance of social participation in providing treatments for individuals with depression, healthcare professionals, including occupational therapy practitioners, need to update their knowledge on current research evidence and effective practical guidance to improve intervention programs for promoting social participation among adults with depression.

The objective of this research was to review intervention programs that support the social participation of adults with depression and their effectiveness. This systematic review only included research studies of nonpharmacological treatment that resulted in behavioral and emotional changes in social participation for the primary focus and also included other effective factors resulting from the intervention programs such as life satisfaction and QoL as relevant to achieve the objective.

## 2. Methods

This systematic review followed the guideline of the Preferred Reporting Items for Systematic Reviews and Meta-Analyses (PRISMA) [19] and focused on nonpharmacological treatment to improve social participation of adults with depression. Research articles involving hospital and community rehabilitation services were included. The researchers considered the studies with Sackett's evidence-based medicine levels I, II, and II [20, 21] and the Oxford Centre for Evidence-Based Medicine (OCEBM) [22] (Table 1). Although randomized control trial (RCT) studies have been accepted as the gold standard of study design to examine the effects of an intervention [23], this study considered setting an eligibility criteria for participants that might influence the response of an intervention program; for example, age, diagnosis, and severity of physical movement. This review followed the definition of social participation provided by the “Occupational Therapy Practice Framework: Domain and Process 4th Edition” [5], which means covering desired participation in community and family activities or involving peers and friends or any subset of activities that relate to social issues, including social participation through remote technologies. This definition was used in selecting the key terms for the search strategy. Thus, the screening process included articles within the occupational therapy, physical therapy, psychotherapy, medicine/psychiatry, and nursing disciplines. This systematic review was registered on PROSPERO 2019 (CRD42019135525).

### 2.1. Search Strategy

The Patient/Problem, Intervention, Comparison, Outcome (PICO) method [19, 24] was used to formulate research questions. Participants were persons with depression, the intervention was nonpharmacological treatment, the comparison was not applicable, and the outcome was social participation (behavioral and emotional change). The researchers searched and recorded the number of studies in the searching step. Literature search was conducted across five electronic databases: PubMed, CINAHL, Wiley Online Library, OTSeeker, and PsycINFO. To further ensure that studies were not left out, the researchers carried out additional hand searches by reviewing the table of contents on the sites of four occupational therapy journals: *American Journal of Occupational Therapy* (AJOT), *British Journal of Occupational Therapy* (BJOT), *Scandinavian Journal of Occupational Therapy* (SJOT), and *Occupational Therapy in Mental Health* (OTMH).

### 2.2. Inclusion and Exclusion Criteria

The inclusion criteria in the initial stage of screening were peer-reviewed scientific articles on adults with depression aged 20 to 60 years old, published in English between January 2010 and December 2018. Searching articles and hand searches were completed in one month. This review included only journal articles and excluded non-peer-reviewed research literature, presentations, conference proceedings, and dissertations. The researchers only considered intervention programs directly provided to persons with depression. The research team considered studies concerning persons with depression and physical comorbidity for inclusion as these individuals face many barriers to social participation. All documents and information were logged and validated by the researchers (SP, RS, AT, JJ, SA, and TS).

The exclusion criteria were as follows: (1) study with other groups of participants unrelated to the purpose of this research, which consisted of (1.1) animal or drug study, (1.2) development of assessment tool, (1.3) family, caregiver, stakeholder, and other groups (e.g., occupational therapy practitioner or students), (1.4) nonspecific participants (e.g., severe mental illness or psychosis), and (1.5) individuals aged <20 or >60 years; (2) different research types of evidence, such as (2.1) qualitative study and (2.2) studies not categorized as evidence-based medicine levels I–III; (3) social participation not being the central aspect in the research; and (4) not an intervention program for persons with depression.

### 2.3. Screening, Eligibility, and Data Extraction

Studies were searched and identified independently through databases by SP and TS, using sets of key terms, and additional hand searches were conducted by SP and JJ. Retrieved studies underwent title and abstract screening, performed independently by SP and JJ. Studies were dismissed at this stage if they did not match the screening criteria, as were the studies with social participation defined remarkably differently from this. In case of any doubt, SA, who has higher qualification and research experience, made the final decision after thorough deliberation and critical thinking. Reference lists of the studies included were also reviewed to ensure their relevance. Next, SP retrieved the full text of the remaining articles, arranged, categorized, and grouped the studies into a spreadsheet before assessing the strength of evidence and risk of bias. The other researchers double-checked each stage.

### 2.4. Synthesis and Quality of Assessing Risk of Bias

The researchers provided a narrative synthesis of the findings. It was structured around the types of intervention programs, characteristics of the target population, and types of outcomes. The guidelines from the U.S. Preventive Services Task Force [24] was used to critique the strength of the evidence (Table 2). This study examined the risk of bias by using the guideline from the *Cochrane Handbook for Systematic Reviews of Interventions* [25] for nonsystematic reviews and the Assessment of Multiple Systematic Reviews (AMSTAR) for systematic reviews, respectively, with the latter having good development [26], reliability, and construct validity and feasibility [27].

## 3. Results

### 3.1. Search Results

**Figure 1:**
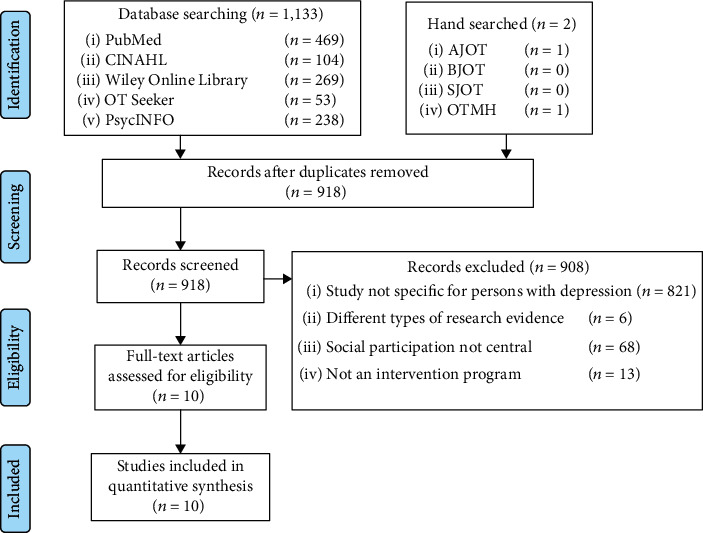
PRISMA flow diagram.

The search found 1,135 studies in the screening process. Among these, ten studies (Berget et al. 2011 [28], Graven et al. 2011 [29], Ammerman et al. 2013 [30], Nagy et al. 2017 [31], Chen et al. 2019 [32], Strøm et al. 2019 [33], Kern et al. 2019 [34], Rogers et al. 2014 [35], Cruwys et al. 2014 [36], Croezen et al. 2015 [37]) were assessed as eligible and were included in the analysis and synthesis (Figure 1). Each study and its details after analysis have been shown in Table 3.

### 3.2. Study Characteristics

In the analysis and synthesis of results, seven of the ten studies (Berget et al. 2011 [28], Graven et al. 2011 [29], Ammerman et al. 2013 [30], Nagy et al. 2017 [31], Chen et al. 2019 [32], Strøm et al. 2019 [33], Kern et al. 2019 [34]) were at level I, while the other three studies (Rogers et al. 2014 [35], Cruwys et al. 2014 [36], Croezen et al. 2015 [37]) were at level III of research levels of evidence. In ten studies, two studies (Berget et al. 2011 [28], Rogers et al. 2014 [35]) were obtained from occupational therapy literature, while the other eight studies were derived from nursing, psychology, and medicine and public health disciplines. The studies from occupational therapy literature included participants diagnosed with not only depression but also other psychiatric problems or symptoms such as posttraumatic stress disorder (PTSD) (Rogers et al. 2014 [35]), schizophrenia, and personality and behavioral disorder (Berget et al. 2011 [28]). Four of the 10 studies categorized the participants into depressive symptoms with comorbidities, such as stroke (Graven et al. 2011 [29]), head and neck cancer (HNC) (Chen et al. 2019 [32]), spine fusion (Strøm et al. 2019 [33]), and obesity (Kern et al. 2019 [34]), while one study cultivated in depressed mothers (Ammerman et al. 2013 [30]).

### 3.3. Intervention Programs

Two major categories of intervention were found with 13 specific programs (Table 4). First, occupational-based intervention (OBI) had six programs. Second, cognitive behavioral therapy-based intervention (CBT-BI) had seven programs.

### 3.4. Effectiveness of the Intervention Programs

These programs sought the following four outcomes: behavioral change of social participation (*n* = 4), reducing depression or depressive symptoms (*n* = 13), life satisfaction (*n* = 4), and quality of life (*n* = 1).

#### 3.4.1. Behavioral Change in Social Participation

The results of four programs showed positive behavioral changes in social participation. First, AAT (Berget et al. 2011 [28]) reflected well on subjective experience toward increased function, interaction, and satisfaction while working with and physically touching animals. Interpersonal communication was shown in increased extroversion and talkative expression, which were associated with self-esteem and coping. Furthermore, the participants' self-reports indicated a pleasurable and uplifting experience in interpersonal communication, which referred to reduced fear of new situations or interaction with other people and promoting an atmosphere of social participation combined with animals as the therapeutic environment. Second, SEP (Rogers et al. 2014 [35]) correlated avoidance with reduced active behavior but supported motivation searching for lively activity. Participating in many sessions of SEP fulfilled the transformative experience. If such programs could create resilience, it truly helped reflect preferred responsiveness to past preferences and experience. Besides, it reinforced the motivation and acceptance of social participation and encouraged social connection through peer relationships, with focus group processing and collaboration among the participants. Surfing was the only sporting reference [35], and no other type of exercise was suggested in the findings of this review.

Third, BCPHE (Chen et al. 2019 [32]) showed progressive physical function from one to three months after participation. Consecutive sessions from the introduction, application of principle into practice, discussion, and reflective feedback from BCPHE may have produced this excellent result [32]. Furthermore, a digital video disc was provided for practicing after the lesson to maintain positive behaviors [32]. Fourth, RA (Croezen et al. 2015 [37]) highlighted growing social participation in the long run by fostering participation after a grandchild's birth or the illness or death of a loved one, such as a child or sibling [37]. RA was arguably at the highest level of social participation than voluntary or charity work, sports and social clubs, educational or training courses, and political or community activities [37].

#### 3.4.2. Depression or Depressive Symptoms

All programs of OBI and CBT-BI presented a reduction of depressive symptoms. Furthermore, SEP (Graven et al. 2011 [29], Nagy et al. 2017 [31], Rogers et al. 2014 [35]), AAT (Berget et al. 2011 [28]), and GBA (Cruwys et al. 2014 [36]) recorded a significant reduction in depression and its symptoms. WBP (Strøm et al. 2019 [33]) and RA (Croezen et al. 2015 [37]) presented a nonsignificant decrease in symptoms during the first follow-up but increased again on subsequent follow-ups at three to six months (Strøm et al. 2019 [33]) or four to five years (Croezen et al. 2015 [37]).

#### 3.4.3. Life Satisfaction

Four programs, SBP, SEP, PS, and GBA from Nagy et al. 2017 [31], showed higher levels of life satisfaction with the social intervention. However, the result was not statistically significant. Life satisfaction in this program covered broader boundaries in receiving a social network, excluding spouse or partner and family relationships.

#### 3.4.4. Quality of Life

SEP (Graven et al. 2011 [29]), WBP (Strøm et al. 2019 [33]), BCPHE (Chen et al. 2019 [32]), and CPG (Cruwys et al. 2014 [36]) studied covering QoL. Each study had different tools to study outcome measurement of QoL. There was QoL (EQ-5D-5L questionnaire) (Strøm et al. 2019 [33]), University of Washington Quality of Life Scale (UW-QoL) (Chen et al. 2019 [32]), and QoL Inventory by Frisch et al. (1992 cited in Cruwys et al. 2014 [36]), while two scales of health-related quality of life (HRQoL) (Stroke Impact Scale-16 item and Short Form-36) were used in two studies of the systematic review (Graven et al. 2011 [29]) along with utilizing best-evidence synthesis (BES) by van Tulder et al. (1999 cited in Graven et al. 2011 [29]). However, only the CPG (Cruwys et al. 2014 [36]) revealed significant improvement in QoL, which is related to decreased depressive symptoms. The BCPHE (Chen et al. 2019 [32]) attempted to measure QoL and its association with physical functioning. Even though the score changed slightly over time in the study, physical limitations still affected the participants' ability to communicate and express social function and QoL. The other programs had insufficient information supporting QoL.

#### 3.4.5. Strength of Evidence

A strong level of certainty was found in seven studies (Berget et al. 2011 [28], Graven et al. 2011 [29], Ammerman et al. 2013 [30], Nagy et al. 2017 [31], Chen et al. 2019 [32], Strøm et al. 2019 [33], Kern et al. 2019 [34]) because they were well-designed and well-conducted at level I of RCT and systematic review studies, possessing a strong finding that met their research objectives. A moderate level of certainty was found in the three other studies (Rogers et al. 2014 [35], Cruwys et al. 2014 [36], Croezen et al. 2015 [37]), as they were at level III of research evidence and had a small sample size or needed future research for more clarification on the effectiveness of the treatment programs. All summaries are illustrated in Table 3.

#### 3.4.6. Risk of Bias

The risk of bias was rated for studies individually (Tables 5 and 6). Among eight studies of nonsystematic reviews, five had different risk of bias items used in the assessment. Low risk of selection bias pointed to five studies (Berget et al. 2011 [28], Ammerman et al. 2013 [30], Chen et al. 2019 [32], Strøm et al. 2019 [33], Kern et al. 2019 [34]), while a high risk of selection bias appeared in the three other studies (Rogers et al. 2014 [35], Cruwys et al. 2014 [36], Croezen et al. 2015 [37]). There was a low risk of performance bias in three studies (Berget et al. 2011 [28], Ammerman et al. 2013 [30], Chen et al. 2019 [32]), whereas a high or unclear risk of performance bias was referred to in five studies (Strøm et al. 2019 [33], Kern et al. 2019 [34], Rogers et al. 2014 [35], Cruwys et al. 2014 [36], Croezen et al. 2015 [37]). Three studies (Strøm et al. 2019 [33], Rogers et al. 2014 [35], Cruwys et al. 2014 [36]) had a high risk of detection bias because they could not be blinded for providing purposive treatment, and the five other studies (Berget et al. 2011 [28], Ammerman et al. 2013 [30], Chen et al. 2019 [32], Kern et al. 2019 [34], Croezen et al. 2015 [37]) had an unclear risk of detection bias. Every study reported findings with a low risk of attrition and biased reporting. Finally, two studies of systematic reviews (Graven et al. 2011 [29], Nagy et al. 2017 [31]) had a low risk of bias in all items of assessment.

## 4. Discussion

This review revealed that not every treatment program affects all behavioral changes in social participation, reduces depression or depressive symptoms, improves life satisfaction, and improves QoL. It could not be determined which program was the most effective due to the diversity of participants' programs and characteristics. The programs within the systematic reviews included in this study (SEP (Graven et al. 2011 [29], Nagy et al. 2017 [31]), OSCSP (Nagy et al. 2017 [31]), SBP (Nagy et al. 2017 [31]), GBA (Nagy et al. 2017 [31]), PsyE (Nagy et al. 2017 [31]), PsyT (Nagy et al. 2017 [31]) revealed a strong level of evidence and low risk of bias. AAT (Berget et al. 2011 [28]), IH-CBT+HV (Ammerman et al. 2013 [30]), and BCPHE (Chen et al. 2019 [32]) show a strong level of strength of evidence and a low risk of bias in some items. AAT (Berget et al. 2011 [28]), however, displayed a convincing behavioral and emotional change as the primary outcome compared with BCPHE (Chen et al. 2019 [32]) and RA (Croezen et al. 2015 [37]). Other programs claim to be useful for at least one element of the outcome. WBP (Strøm et al. 2019 [33]) influenced less effectiveness in diminishing depression. Nevertheless, the virtual program might facilitate social interaction in cases of one who needs appropriate social cues or responsiveness with nonbehaviors, particularly severe physical condition. Thus, this program needs further study to confirm behavioral changes in social participation, especially using a more appropriate assessment tool.

AAT (Berget et al. 2011 [28]), SEP (Graven et al. 2011 [29], Nagy et al. 2017 [31], Rogers et al. 2014 [35]), RA (Croezen et al. 2015 [37]), GBA (Nagy et al. 2017 [31], Cruwys et al. 2014 [36]), SBP (Nagy et al. 2017 [31]), and OSCSP (Nagy et al. 2017 [31]) are occupation-based interventions that challenged and coached participants by receiving direct and indirect feedback from their activity and participation. AAT (Berget et al. 2011 [28]) may improve confronting and dealing with frustration between pleasant and ambivalent feelings when participating socially [38]. As surfing was mentioned in the effective SEP (Rogers et al. 2014 [35]), general types of exercise, such as walking, football, and yoga (Nagy et al. 2017 [31]) did not show significant effective findings of reviewing. However, several sports or exercises were accepted in the literature as beneficial treatments for people with depression [39–43], such as weightlifting [39], aerobic exercise [40], and walking [39]. Thus, these two programs are highly recommended for improving social participation in adults with depression. The RA enabled social participation, spirituality, and mental health through religious observance as an occupation [44, 45]. Even though there was no report on the frequency and direction in the mechanism of behavior in the participation, it helped in uplifting mood [37] as active religious persons might protect themselves from depression by receiving social attachment [45] and promoting a coping mechanism [46]. On the other hand, a study argued that participating in religious activity may enhance feelings of guilt or discouragement, depending on tradition [46]. This review concluded that this program, which promoted social participation, is associated with declining depression over a long period of time, even though it was at the moderate level of certainty and had high and unclear risk of bias. Thus, these programs go against depression resulting in participation promoted by creating real supportive physical and social environments. Therefore, they can be useful training programs for improving mental health in independent living [8, 47]. Furthermore, the importance of these treatment programs, from cultivated evidence, should be highlighted more and integrated into occupational therapy practice to promote expressive behavioral change in social participation and encourage self-love, self-worth, and a sense of belonging. Meanwhile, these programs promote mental and social health as stimulation for physical health and well-being.

CPG (Cruwys et al. 2014 [36]), WBP (Strøm et al. 2019 [33]), BCPHE (Chen et al. 2019 [32]), BADLI (Kern et al. 2019 [34]), IH-CBT+HV (Ammerman et al. 2013 [30]), PsyE (Nagy et al. 2017 [31]), and PsyT (Nagy et al. 2017 [31]) were tied basically to the study of psychology and the social learning theory in supportive psychoeducation, CBT, and behavioral activation (BA). They increased the familiarity of the activity and shifted decision-making from being an obstacle-focused to participation-focused [36]. This review could not ignore the benefit of CBT programs in the intervention of social participation, as they integrate suitably into the treatment program for depression, in order to obtain self-efficacy and personal performance, especially in shifting decision-making, which is an obstacle in developing social participation in the initial stage of nonpharmacological treatment. Thus, these programs help to establish a visible behavioral change in social participation and affirm the programs at the strong level of certainty and low risk of bias, especially in the study of the behavioral change program [32]. Health care practitioners can use these programs to impart social support by matching individual goals with social needs to improve treatment responsiveness and develop social skills [48] that possibly advocate QoL and prevent the risk of depression relapse. The application of treatment in social participation programs occurs in a real environment. Hence, there is a merit in integrating community-based rehabilitation (CBR) and CBT into these programs to improve social participation, life satisfaction, and QoL among adults with depression. OSCSP (Nagy et al. 2017 [31]), SEP (Graven et al. 2011 [29], Nagy et al. 2017 [31]), SBP (Nagy et al. 2017 [31]), GBA (Nagy et al. 2017 [31]), PsyT (Nagy et al. 2017 [31]), and PsyE (Nagy et al. 2017 [31]) were arranged recently in CBR promotion since having CBR in promoting social participation treatment programs [48, 49] contributes to mental health not only for people with depression but also in creating good social support for their family and community members.

Strong evidence was presented in most programs; however, an essential factor of the considerable rating level of certainty depended on whether their assessment tools were suitable or further study was needed. The assessment risk of bias was low in systematic review studies. The studies were considered as having nonsystematic reviews if they had a high risk of bias, except for some RCT studies. However, some parts of the RCT studies had a high risk of bias regarding advantages for the participants because they could not design the study with blinded personnel in treatment and outcome measurement. This systematic review provides a comprehensive appraisal of the effectiveness of intervention programs that support social participation for adults with depression and advocate the necessity of occupational therapy and healthcare services in the nonpharmacological treatment for depression in both clinical and community settings.

## 5. Limitation

This review gathered and classified the intervention programs to support social participation in adults with depression; however, this study's limitations should also be considered. Accordingly, the researchers focused more on nonpharmacological treatment in support of social participation and evidence in occupational therapy research, and several articles showed limitations after screening. Expanding databases and years of searching relate to a change of results when reviewing. Interpretation of the findings from several intervention programs was carefully performed because of the variation in examining intervention, diagnosis, and age range of the participants. The age of participants was accepted if the articles concerned only adolescents aged 16 years and above [30], 18 years and over [34, 35], or adults aged 50 years and more [37]. Thus, this research reviewed the reviewing structure, and the results met the research objective. As a suggestion for further research, participants could be extended to adolescents or older people, which may show various activities or treatment programs of social participation. Most of the articles in this study seemed to have unclear confirmation of behavioral change expression in social participation due to the use of self-report assessment tools and limited findings in elucidating life satisfaction and QoL. Thus, future studies can focus on identifying these issues and how they work. When promoting social participation in a group of people with depression, the implications of this study may raise awareness on the matter.

## 6. Conclusion

This study reviewed the evidence of nonpharmacological treatments that illustrate intervention programs and their effect on social participation for adults with depression. Programs were categorized as OBI or CBT-BI and summarized in order to demonstrate the effectiveness of interventions. Four programs (AAT, SEP, RA, and BCPHE) demonstrated visible behavioral changes in social participation. All intervention programs reported decreasing depressive symptoms. Four intervention programs (SBP, SEP, PS, and GBA) illustrated life satisfaction, whereas only CPG promoted QoL. Based on the result of this review, AAT, SEP, and BCPHE are recommended. A combined treatment with a flexible and suitable application for covering higher benefits in promoting social participation, reducing depression, and contributing to life satisfaction and QoL is recommended for other programs.

## Figures and Tables

**Table 1:** The level of evidence.

Level	Type of evidence
Level I	Systematic reviews, meta-analyses, randomized controlled trials
Level II	Two groups, nonrandomized studies (e.g., cohort, case-control)
Level III	One group, nonrandomized (e.g., before and after, pretest and posttests)
Level IV	Descriptive studies that include analysis of outcomes (single-subject design, case series)
Level V	Case reports and expert opinion that include narrative literature reviews and consensus statements

Adapted from Sackett's evidence-based medicine [20, 21] and the Oxford Centre for Evidence-Based Medicine (OCEBM) [22].

**Table 2:** Strength of evidence (level of certainty).

Strength	Description
Strong	(i) Two or more level I studies (ii) The available evidence usually includes consistent results from well-designed, well-conducted studies. The findings are strong, and they are unlikely to be strongly called into question by the results of future studies
Moderate	(i) At least one level I high-quality study or multiple moderate-quality studies (level II, level III, etc.) (ii) The available evidence is sufficient to determine the effects on health outcomes, but confidence in the estimate is constrained by such factors as (i) the number, size, or quality of individual studies (ii) inconsistency of findings across individual studies As more information (other research findings) becomes available, the magnitude or direction of the observed effect could change, and this change may be large enough to alter the conclusion related to the usefulness of the intervention
Low	(i) Small number of low-level studies, flaws in the studies, etc. (ii) The available evidence is insufficient to assess effects on health and other outcomes of relevance to occupational therapy. Evidence is insufficient because of the following: (i) The limited number or size of studies (ii) Important flaws in study design or methods (iii) Inconsistency of findings across individual studies (iv) Lack of information on important health outcomes More information may allow estimation of effects on health and other outcomes of relevance to occupational therapy

Reference: the U.S. Preventive Services Task Force [24].

**Table 3:** Study characteristics, activities or treatment program, and strength of evidence.

Author/Ref	Level of evidence/research design	Participants, age ranges, and sample size	Outcome measures	Activity or treatment program	Result	Strength of evidence
Berget et al. 2011 [28]	Level I/RCT	*Included participants*: various diagnoses (*n* = 90), F30-39 (*n* = 22), age 34.7 ± 10.7 years, both in- and outpatients *Completed participants*: Treatment (*n* = 41), control (*n* = 28)	Score change; SB, SA, SSMA in BDI & STAI	*Intervention program*: 12-week AAT with farm animals (dairy cows, sheep, horses, meat production with cattle) Main tasks: patting, brushing, washing, saddling, riding horses, and moving animals from different places in the cowshed and between different pastures *Control group*: usual treatment	Depression significantly decreased between baseline and six-month follow-up in both treatment and control groups, but no significant difference was observed in depression scores between the treatment and control group at any point in time. Patients with the largest reduction in BDI scores reported the largest increase in coping (*p* = .0006), mood (*p* = .049), self-esteem (*p* = .004), and extroversion (*p* = .06). Favoring physical contact with the animals correlated strongly with improved mood.	Strong
Graven et al. 2011 [29]	Level I/systematic review	RCT articles only *Databases*: Medline (*n* = 241), Cochrane Library (*n* = 282), CINAHL (*n* = 1,086), PEDro (*n* = 755) Satisfied criteria (*n* = 71) Final included (*n* = 54)	PEDro scale rating Meta-analysis and BES Primary outcome: physical ability and basic ADL (*n* = 54) Secondary outcome: depressive symptoms, participation, QoL (*n* = 29)	Group 1: compared intervention group to usual care or placebo control (*n* = 36) Group 2: compared two interventions. At times, difficult to ascertain whether the control condition was placebo control or alternate intervention (*n* = 18) For the study's 2^nd^ outcome, the control group may have been described as a placebo in terms of primary outcome but may have had a treatment effect in secondary outcomes	A total of 54 studies were classified into nine types. Three types showed related findings, but only exercise showed an effective outcome. (1) Exercise: this had a significant reduction in depression at three months' follow-up for those who had a baseline depressed mood score. But insufficient evidence for improving participation or QoL and promote home-based and physical rehabilitation exercise. (2) Single discipline community-based rehabilitation: insufficient to limited evidence in improving depressed mood, participation, and QoL aspects. (3) Care coordination, psychosocial and interdisciplinary management: no BES classification possible for depressed mood, participation, and QoL.	Strong
Ammerman et al. 2013 [30]	Level I/RCT	*Participants:* mothers with MDD (*n* = 93), mean age 21.9 ± 4.8 Randomized in IH-CBT (*n* = 47), SHV (*n* = 46) Completed 15 sessions + 1 month posttest = 48.9%	Measure at pre-, post-, and three-month follow-up treatments EPDS SCID-I BSI ISEL SNI	*Intervention program*: IH-CBT and home visiting; IH-CBT: 15 sessions, weekly, +lasted 60 mins plus a booster session one-month posttreatment. Home visiting consisted of HFA (*n* = 81) and NFP (*n* = 12) *Control group*: standard home visit (SHV)	Psychological distress decreased (broad improvement) at posttreatment and follow-up. IH-CBT increased social support (affiliative and belongingness aspects), whereas tangible support was not significant. No group differences were found in size and involvement with social networks.	Strong
Nagy et al. 2017 [31]	Level I/systematic review	*Databases*: Cochrane Database, Medline, Embase, PsycInfo, CINAHL, TRoPHI Published Jan 1995–Oct 2014 24 studies met inclusion criteria and studied in community settings	WMHCIDI BDI-Center for Epidemiologic Studies Depression Scale Assessment risk of bias	(1) Peer support, e.g., sharing and empathizing with others (2) Skill-building, e.g., coping skills, action planning skills (3) Group-based activities, e.g., team building activities, community clubs, outing within the community, group walk, horticultural activities, trust-building task (4) Psychoeducation, e.g., group-based education on contributors to stress, depressive symptoms, and mental well-being (5) Psychotherapy, e.g., cognitive behavioral therapy and interpersonal therapy (6) Exercise, e.g., walking, playing football, yoga (7) Linking community resources, e.g., linking participants with various supports and resources in the community	22 of 24 studies used a combination of approaches. 17 of 24 studies showed a reduction in depressive symptoms. One study (treatment: peer support, group-based activities, exercise, and skill building) reported improved life satisfaction. Most RCT studies had a low risk of bias, but several indicators were unclear toward less information. In comparison, the nonrandomized study had greater variability resulting in a risk of bias.	Strong
Chen et al. 2019 [32]	Level I/RCT	*Participants*: outpatient (*n* = 100), age ≥ 20 years old with diagnosis of head and neck cancer. Experimental group (*n* = 50), control group (*n* = 50)	Four-time score measure; baseline (T0)—after the program (T1, T2, T3) HADS LSAS UW-QoL KPS	*Intervention program*: (1) Educational manual for personal hygiene treatment, principal and skill social interaction, and supportive psychological care (2) BCPHE; 40 mins, five sessions/day (3) Introduction (4) Application principles and skills (3) Individual verbal and nonverbal behaviors to communication and interaction (4) Group discussion, reflection, and feedback (5) Questions and answers *Control group*: routine care	Less fear of social interactions, less avoidance of social interactions, and improved physical function during the three months after the intervention.	Strong
Strøm et al. 2019 [33]	Level I/RCT	*Participants*: lumbar spine fusion with depressive symptoms (*n* = 99), age ≥ 18 years Intervention group (*n* = 48), control group (*n* = 51)	HADS ODI QoL (EQ-5D-5L questionnaire) LBPRS	*w-SPIINA*: animation displayed chronology of initial preparation for surgery to postsurgical rehabilitation for the first three months at home ISG and a diary visualizing the progress in pain and activity to increase patients' satisfaction with social life *Control group*: standard treatment	No significant difference within the treatment and the control group regarding changes in HADS at three-month follow-up. No significant differences between groups (outcome measures: symptoms of anxiety and depression, pain, disability, and QoL). The depressed scores declined in cases of both anxiety and depression from baseline to three-month follow-up before they increased again from 3 to 6 months.	Strong
Kern et al. 2019 [34]	Level I/RCT	*Participants*: women MDD with obesity (*n* = 78), age 18–65 years Intervention group, mean age 45.6 ± 10.9 Control group, not specified	BDI-II weekly, during the baseline, six-month follow-up assessments Social impairment and goal-directed behavior using the BA for Depression Scale (BAD)	*Intervention program:* be active condition, individual delivered BAD then a group-based lifestyle intervention Standard condition: group-based lifestyle intervention Both conditions had intensive treatment and maintenance phase, six months each	Greater improvement in hedonic capacity, environmental reward, and social impairment was associated with greater reductions in depression over six months.	Strong
Rogers et al. 2014 [35]	Level III/pre- and posttest	*Participants*: outpatient (*n* = 14), PTSD (*n* = 11), depression (*n* = 1), both (*n* = 2) Age: <24 years (*n* = 3), 24–30 years (*n* = 10, >30 years (*n* = 1) Completed baseline and follow-up (*n* = 11), attended ≥3 sessions (*n* = 10)	Brief self-report questionnaire The PTSD Checklist- Military version Major Depression Inventory (self-report)	Sports-oriented intervention using surfing in an experiential and skill-based program Sessions combine the active experience of surfing with the focused group processing and collaborative social participation among civilian volunteers and fellow veterans 25–35 persons/group; 4 hours/session, five sessions/five weeks	Clinically meaningful improvement in PTSD severity (*p* = .0005) and depressive symptoms (median scores decreased from 33 to 14, *p* = .028). Decreasing depression and reducing PTSD symptom clusters: symptom clusters, avoidance, and hyperarousal but no change in intrusion symptoms.	Moderate
Cruwys et al. 2014 [36]	Level III/two longitudinal intervention studies	*Participants, study 1*: community members' risk of depression (*n* = 52), mean age 44.65 ± 13.79 *Participants, study 2*: outpatients completed CBT (*n* = 92); depression (*n* = 48), anxiety (*n* = 44), mean age 44.75 ± 12.86	*Study 1*: DASS-21, social identification, frequency of attendance *Study 2*: symptom checklist; ZSRDS, BAI, QoL Inventory Social identification	*Study 1: community reintegration group*: joining 1 of 4 recreational, social groups (“Reclink”) run by a community organization; indoor soccer, sewing, yoga, art, at least monthly *Study 2: clinical psychology group*: interventions focused on learning new cognitive and behavioral skills and involved active participation during sessions and homework tasks 2–3.5-hour groups per week, four weeks with groups of 6–12 patients	Both treatment programs contributed to a decline in depression. In study 1: program provided benefit from the social group as a group member. Social identification is expected to benefit from a community-based intervention to reduce social isolation. In study 2: benefits of social identification were greater for depressive symptoms, and it showed significant improvement in the quality of life.	Moderate
Croezen et al. 2015 [37]	Level III/one group longitudinal study	*Participants* from 12 countries, 1^st^ wave (2004/2005) (*n* = 31,115), followed by 2^nd^ wave (2006/2007) and wave 4^th^ (2010/2011) (*n* = 10,706) Aged ≥50 years, mean age = 63 Excluded 3^rd^ wave, not assessed depressive symptoms	Depressive symptom (EURO-D scale) Focus mainly on changes between wave 1 and 2	Study activities: (1) Voluntary or charity work (2) Educational or training courses (3) Sports, social clubs, or other kinds of club activities (4) Participation in religious organizations (5) Participation in political or community organizations	The prevalence of depressive symptoms declined between waves 1 and 2 but increased between waves 2 and 4. Increased participation in religious activities was associated with a decline in depressive symptoms, whereas increased participation in political/community organizations predicted higher depressive symptoms score.	Moderate

Abbreviation: ADL: activity of daily living; AAT: animal-assisted therapy; BA: behavioral activation; BAI: Beck Anxiety Inventory; BCPHE: behavior change program and health education; BDI: Beck Depression Inventory; BES: best evidence synthesis; BSI: Brief Symptom Inventory; CBT: cognitive behavioral therapy; DASS: Depression Anxiety Stress Scales; EPDS: Edinburgh Postnatal Depression Scale; HADS: Hospital Anxiety and Depression Scale; HFA: Healthy Families America; HNC: head and neck cancer; IH-CBT: in-home cognitive behavioral therapy; ISEL: Interpersonal Support Evaluation List; ISG: Internet Support Group; KPS: Karnofsky Performance Scale; LBPRS: low back pain rating scale; LSAS: Liebowitz Social Anxiety Scale; MDD: major depressive disorder; NFP: nurse-family partnership; ODI: Oswestry disability index; PEDro: Physiotherapy Evidence Database; PLF: instrumented posterolateral fusion; PTSD: posttraumatic stress disorder; QoL: quality of life; SA: score after; SB: score before; SCID-I: Structured Clinical Interview for DSM-IV Axis I Disorders, January 2007 version; SHV: standard home visiting; SNI: Social Network Index; SSMA: score six months after; STAI: the Spielberger State Anxiety Inventory; UW-QoL: University of Washington Quality of Life Scale; WMHCIDI: World Mental Health Composite International Diagnostic Interview; w-SPIINA: web-based spine platform featuring interaction and information by animation; ZSRDS: Zung Self-Rating Depression Scale.

**Table 4:** Two major categories of intervention.

Programs	Authors
Occupational-based intervention (OBI)	
Animal-assisted therapy (AAT)	Berget et al. 2011 [28]
Sport or exercise program (SEP)	Graven et al. 2011 [29], Nagy et al. 2017 [31], Rogers et al. 2014 [35]
Religious activity (RA)	Croezen et al. 2015 [37]
Group-based activity (GBA)	Nagy et al. 2017 [31], Cruwys et al. 2014 [36]
Skill-building program (SBP)	Nagy et al. 2017 [31]
Other social and community supportive program (OSCSP), which covers peer support (PS) and linking community resource (LCR)	Nagy et al. 2017 [31]
Cognitive behavioral therapy-based intervention (CBT-BI)	
Web-based program (WBP)	Strøm et al. 2019 [33]
Clinical psychotherapy group (CPG)	Cruwys et al. 2014 [36]
Behavioral change program and health education (BCPHE)	Chen et al. 2019 [32]
In-home cognitive behavioral therapy and home visit (IH-CBT+HV)	Ammerman et al. 2013 [30]
Behavioral activation for depression and lifestyle intervention (BADLI)	Kern et al. 2019 [34]
Psychoeducation (PsyE)	Nagy et al. 2017 [31]
Psychotherapy (PsyT)	Nagy et al. 2017 [31]

**Table 5:** Risk-of-bias table for considering nonsystematic reviews.

Citation	Selection Bias		Performance bias	Detection bias		Attrition bias	Reporting bias
	Random sequence generation	Allocation concealment	Blinding of participants and personnel	Blinding of outcome assessment: self-reported outcomes	Blinding of outcome assessment: objective outcomes	Incomplete outcome data	Selective reporting
Berget et al. 2011 [28]	+	+	+	?	?	+	+
Ammerman et al. 2013 [30]	+	+	+	?	?	+	+
Chen et al. 2019 [32]	+	+	+	?	?	+	+
Strøm et al. 2019 [33]	+	—	—	—	—	+	+
Kern et al. 2019 [34]	+	—	?	?	?	+	+
Rogers et al. 2014 [35]	—	—	—	—	—	+	+
Cruwys et al. 2014 [36]	—	—	—	—	—	+	+
Croezen et al. 2015 [37]	—	—	—	?	?	+	+

Categories for risk of bias: +: low risk of bias; ?: unclear risk of bias; –: high risk of bias; NA: not applicable. Risk-of-bias table format followed the guideline from *Cochrane Handbook for Systematic Reviews of Interventions* by Higgins J.P.T., Altman D.G., and Sterne J.A.C., version 5.1.0 (updated March 2011) [25].

**Table 6:** Risk-of-bias table for considering systematic review (AMSTAR).

Citation	(1)	(2)	(3)	(4)	(5)	(6)	(7)	(8)	(9)	(10)	(11)
Graven et al. 2011 [29]	+	+	+	+	+	+	+	+	+	+	+
Nagy et al. 2017 [31]	+	+	+	+	+	+	+	+	+	+	+

Note 1. Categories for risk of bias: +: low risk of bias; ?: unclear risk of bias; –: high risk of bias; NA: not applicable. Risk-of-bias table format followed the Development of AMSTAR by Shea et al. [26] and developing reliability and validity of AMSTAR by Shea et al. [27]. Note 2. (1) “a priori design” included? (2) Duplicate study selection/data extraction? (3) Comprehensive literature search performed? (4) Status of publication as inclusion criteria? (5) List of included/excluded studies provided? (6) Characteristics of included studies are provided? (7) Quality of studies assessed and documented? (8) Quality assessment was used appropriately? (9) Methods used to combine results appropriate? (10) Likelihood of publication bias assessed? (11) Conflict of interest stated?

## Data Availability

The reviewing data used to support the findings of this study are included within the article.
